# Decreased ATF5 level contributes to improved mitochondrial function in oocytes exposed to vitrification stress

**DOI:** 10.3389/fcell.2024.1431683

**Published:** 2024-09-20

**Authors:** Guizhen Zhou, Aiju Liu, Jiachen Bai, Hongyu Liu, Yixiao Zhu, Yuwen Luo, Lv Zheng, Yunpeng Hou, Jun Li, Xiangwei Fu

**Affiliations:** ^1^ National Engineering Laboratory for Animal Breeding, Key Laboratory of Animal Genetics, Breeding and Reproduction of the MARA, Beijing Key Laboratory for Animal Genetic Improvement, State Key Laboratory of Animal Biotech Breeding, College of Animal Science and Technology, China Agricultural University, Beijing, China; ^2^ State Key Laboratories of Agrobiotechnology, College of Biological Sciences, China Agricultural University, Beijing, China; ^3^ Department of Reproductive Medicine, Reproductive Medical Center, The First Hospital of Hebei Medical University, Shijiazhuang, China

**Keywords:** mitochondrial unfolded protein responses, ATF5, mitochondrial dysfunction, vitrification, oocytes

## Abstract

**Background:**

Mitochondrial unfolded protein response (mtUPR) plays an essential role in the response of mitochondria to stress-induced damage. Activating of transcription factor 5 (ATF5) can help to sustain mitochondrial function and regulate organelle recovery under mitochondrial stress. Vitrification is a stressor that disrupts mitochondrial activity and cell homeostasis. However, little is known about the function of ATF5 in response to the extreme biophysical and chemical stresses during oocyte vitrification.

**Methods:**

The expression of ATF5 and mtUPR biomarkers were measured in fresh and vitrified oocytes. Subsequently, oocytes with ATF5 deficiency were constructed by siRNA microinjection, and the function of ATF5 in mitochondrial function and oocyte development were analyzed in vitrified oocytes. Furthermore, transcriptome analysis was performed to uncover the molecular network regulated by ATF5 in response to oocyte vitrification.

**Results:**

In the present study, the mitochondrial membrane potential and ATP levels were decreased in ATF5 knockdown oocytes, in line with the phenotypes observed in vitrified oocytes. In addition, ATF5 knockdown resulted in decreased mitochondrial temperature, reduced unfolded protein levels, abnormal mitochondrial dynamics (fusion and fission), and increased autophagy. Subsequent experiments indicated that mtUPR was suppressed in oocytes with ATF5 knockdown. Interestingly, ATF5 was aberrantly upregulated in oocytes exposed to vitrification stress. Reduced ATF5 expression to a homeostatic level in vitrified oocytes led to accumulated unfolded protein levels and increased mitochondrial membrane potential. Moreover, increased mitochondrial dynamics and an increased germinal vesicle breakdown (GVBD) rate were detected after *in vitro* maturation. Transcriptome analysis revealed that ATF5 is involved in the vitrification stress response, and ATF5 regulated the *in vitro* maturation potential in vitrified oocytes through the cAMP-PKA and PI3K/AKT pathways.

**Discussion:**

Our findings indicate that mtUPR was initiated in response to vitrification stimuli, and downregulated ATF5 level to a homeostatic state contributes to improved mitochondrial function in oocytes exposed to vitrification stress. Our results highlight the crucial role of ATF5 in the regulation of mitochondrial function in vitrified oocytes through mediating mtUPR.

## Introduction

Mitochondria are crucial double-membrane organelles that are involved in essential cellular events, such as mitochondrial proteostasis maintenance (Gokhale et al., 2021), mitophagy (Chen, Z. H. et al., 2017), apoptosis (Yee et al., 2014), and mitochondrial stress response (Köhler et al., 2015). Mitochondrial unfolded protein response (mtUPR) is one of the main mitochondrial stress response mechanisms. Evidence has shown that mtUPR plays a crucial role in maintaining mitochondrial homeostasis through stress activated transcription factor atfs-1 (ATFS-1)/activating transcription factor 5 (ATF5)-mediated transcription regulation (Soo et al., 2021; Held et al., 2022). Previous findings have indicated that, in *Caenorhabditis elegans*, mtUPR is predominantly mediated by the transcription factor ATFS-1 (Nargund et al., 2012). Under normal conditions, ATFS-1 is normally transported into mitochondria and degraded by the mitochondrial protease Lon (Shao et al., 2016). When exposed to mitochondrial stress, ATFS-1 accumulates in the cytoplasm and enters into the nucleus, where it activates the mtUPR (Kaufman and Crowder, 2015; Soo et al., 2021). It is noteworthy that ATF5 has been identified as a mammalian equivalent of ATFS-1, and could indeed induce mtUPR in *C. elegans* lacking ATFS-1 (Haynes et al., 2010; Fiorese et al., 2016). Studies have shown that ATF5 improves intestinal barrier function via a metabolically mediated mtUPR regulation mechanism (Chamseddine et al., 2022). Furthermore, ATF5 is also required for organelle recovery after mitochondrial stress in mammalian cells (Fiorese et al., 2016).

Efficient oocyte cryopreservation is fundamental for successful fertility preservation and the wider application of assisted reproductive techniques (ART). Over the past few decades, cryogenic freezing technology has developed rapidly, both at home and abroad (Li et al., 2021). Compared to the slow freezing method, vitrification is much more prevalent due to its relatively simple procedure, low cost, and high survival rate. However, vitrification causes disrupted mitochondrial function and can dramatically decrease the quality of oocytes (Lan et al., 2022). A previous study showed that mitochondria were highly susceptible to damage caused by temperature, osmolarity, and ice crystal formation during freezing and thawing (Zhang et al., 2016). A growing body of evidence has indicated that vitrification mainly causes damage to the mitochondria, which ultimately leads to oxidative stress, autophagy, and cell apoptosis (Zhuan et al., 2022). Notably, aggregated mitochondria were observed in the central area in oocytes after vitrification (Lee et al., 2021). As mitochondria play key roles in the development of oocytes, identifying the mechanism responsible for mitochondrial disturbances would be pivotal to improve the efficiency of oocyte vitrification.

It has been reported that the accumulation of oxidatively damaged or unfolded proteins in mitochondria leads to mitochondrial stress which, in turn, activates the mtUPR signaling pathway to alleviate the damage (Sheng et al., 2022). Studies have also suggested that the mitochondrial chaperone protein HSP60 can be used as an indicator for the occurrence of mtUPR (Keerthiga et al., 2021; Zhou et al., 2021). During mitochondrial dysfunction and oxidative stress injury, SIRT1 may control ATF5 to rescue injured mitochondria through mtUPR (Shi et al., 2024). At the same time, mtUPR damage accelerated the shortening of mouse oocyte telomeres, leading to embryonic development failure and sterility (Ergun et al., 2024). However, the incidence of mtUPR and the role that it plays in mammalian oocytes under external stresses remain unexplored. Therefore, the effect of ATF5 on oocyte mitochondrial function under vitrification stimuli was systematically investigated for the present study, with the aim of providing novel insights related to the critical role of mitochondrial proteostasis in maintaining oocyte development.

## Results

### Atf5 knockdown impaired mitochondrial function in oocytes

**FIGURE 1:**
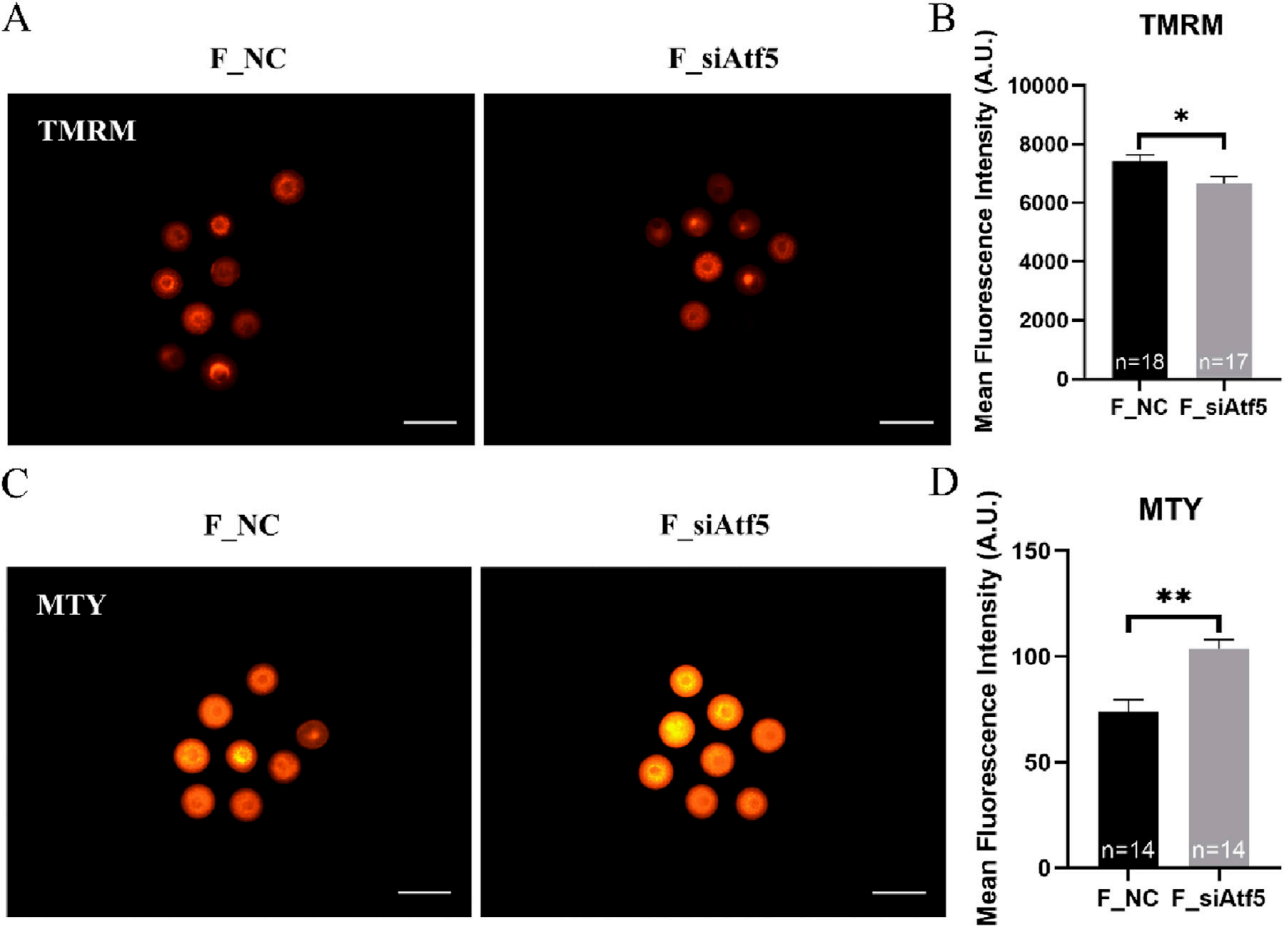
Atf5 knockdown impairs potential of the mitochondrial membrane and mitochondrial temperature in GV stage oocytes. **(A, B)** Potential of the mitochondrial membrane after Atf5 knockdown. Scale bar, 100 μm. **(C, D)** Mitochondrial temperature probe MTY fluorescent staining and fluorescent intensity analysis upon Atf5 knockdown. Scale bar, 100 μm. All experiments were performed with at least three biological replicates and the data represent the means ± SEMs. *P < 0.05, **P < 0.01, ns = no significance.

**FIGURE 2:**
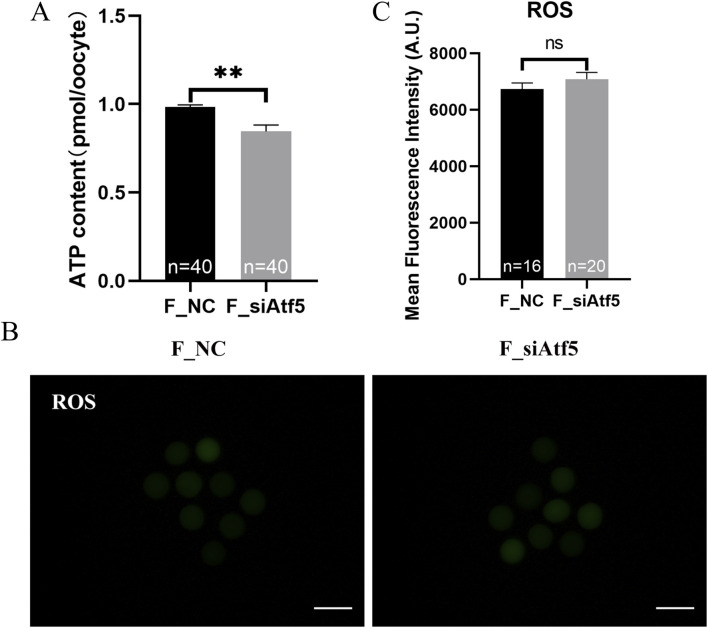
Atf5 knockdown affects ATP levels and impairs ROS levels in GV stage oocytes. **(A)** ATP levels after Atf5 knockdown. **(B, C)** ROS levels after Atf5 knockdown. Scale bar, 100 μm. All experiments were performed with at least three biological replicates and the data represent the means ± SEMs. *P < 0.05, **P < 0.01, ns = no significance.

**FIGURE 3:**
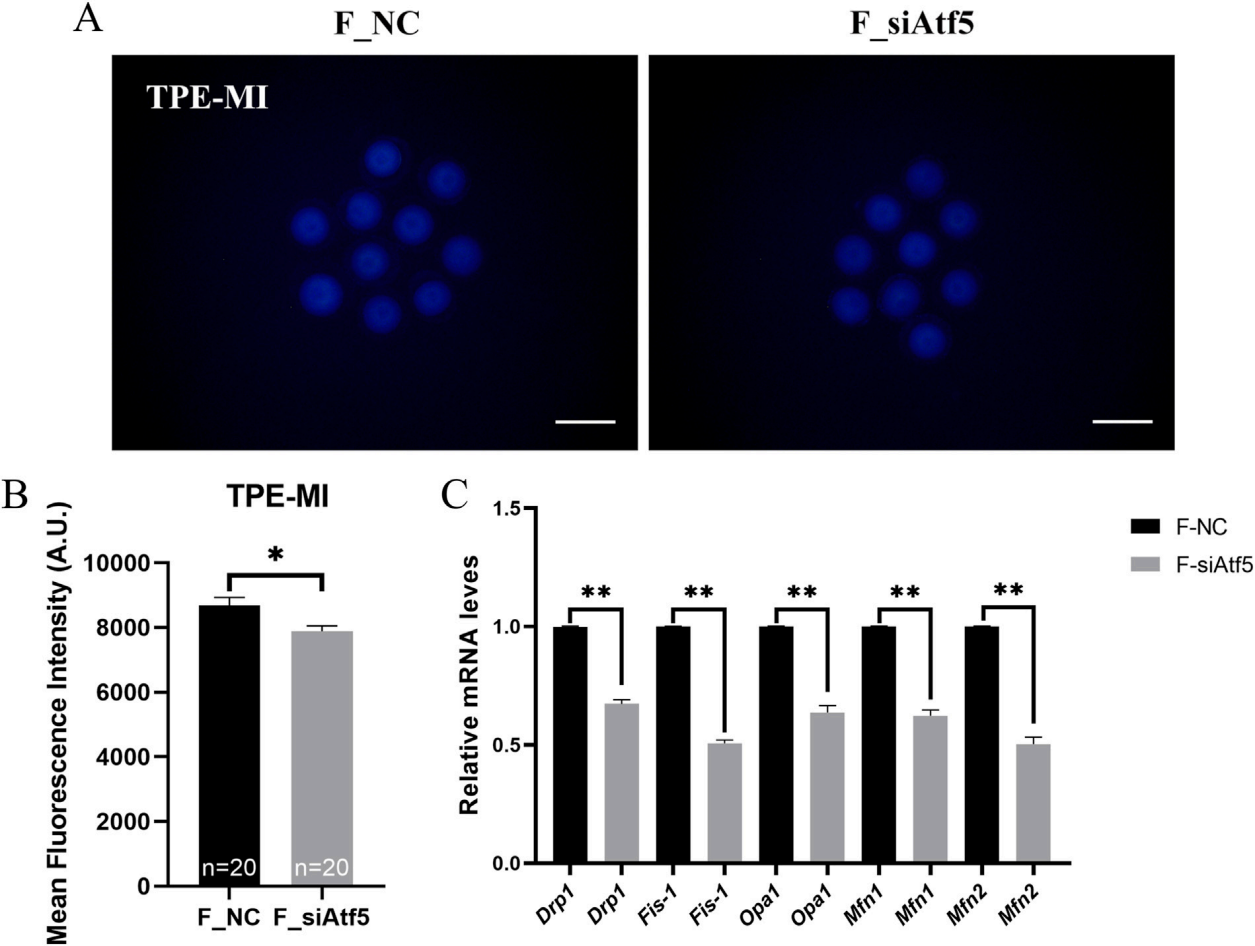
Atf5 knockdown affects the unfolded proteins and mitochondrial dynamics in GV stage oocytes. **(A, B)** TPE-MI dye can be used to detect the unfolded proteins after Atf5 knockdown. Scale bar, 100 μm. **(C)** The relative expression level of mitochondrial dynamic gene mRNA in F_NC vs. F_si. All experiments were performed with at least three biological replicates and the data represent the means ± SEMs. *P < 0.05, **P < 0.01, ns = no significance.

Mitochondria provide energy to the cell and play a crucial function in oocyte development. Therefore, we examined mitochondrial function alterations after Atf5 knockdown by fluorescence staining and microplate reader detection. Significant declines in mitochondrial membrane potential (7410.00 ± 217.20 vs. 6664.00 ± 230.50, *P* < 0.05; Figures 1A, B), mitochondrial temperature (73.93 ± 5.58 vs. 103.70 ± 4.33, *P* < 0.01; Figures 1C, D), and ATP levels (0.98 ± 0.01 vs. 0.85 ± 0.04, *P* < 0.01) were observed in the F_si group (Figure 2A), whereas the ROS level did not significantly differ (6728.00 ± 219.30 vs. 7087.00 ± 237.80, *P* > 0.05; Figures 2B, C). A previous study has indicated that unfolded proteins in the mitochondria activate mtUPR, which exhibits a protective role in the maintenance of protein folding homeostasis (Korennykh and Walter, 2012). We used TPE-MI probes to evaluate the expression profile of unfolded protein levels in the oocytes. Our results indicated that the unfolded protein levels were significantly decreased in the F_si group (8681.00 ± 245.30 vs. 7889.00 ± 164.60, *P* < 0.05; Figures 3A, B). These findings indicate that Atf5 knockdown induced mitochondrial dysfunction and a decrease in unfolded protein content.

Mitochondrial dynamics (fusion and fission) are required for a wide range of mitochondrial function, including autophagy, cellular stress responses, and apoptosis (Wang et al., 2020). Meanwhile, a previous study has indicated that ATF5 is involved in autophagy and apoptosis regulation (Dluzen et al., 2011; Sheng et al., 2011). Thus, the effect of ATF5 on mitochondrial dynamics, autophagy, and apoptosis was investigated.

**FIGURE 4:**
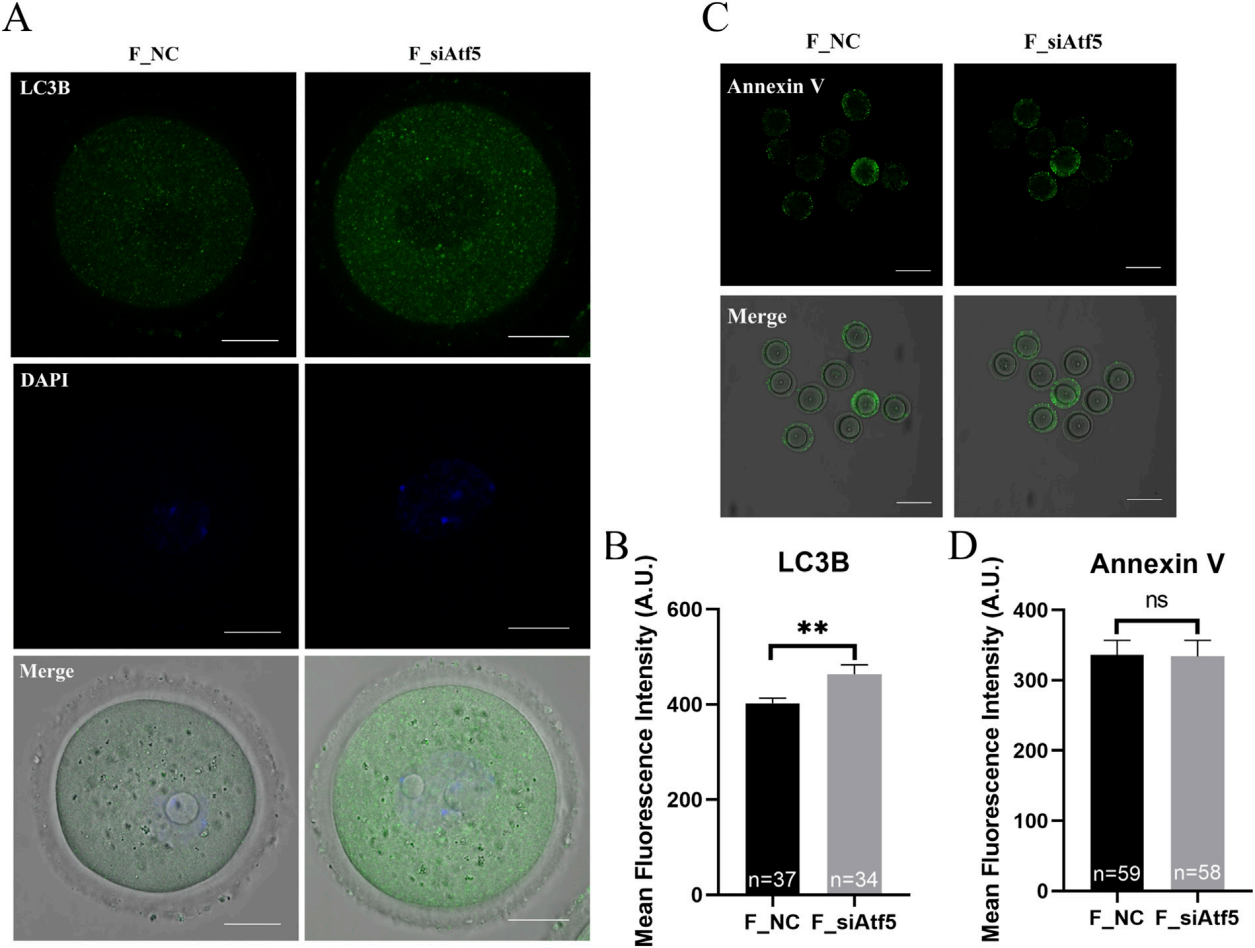
Atf5 knockdown affects autophagy and early apoptosis in GV stage oocytes. **(A)** Fluorescence staining of autophagy. Scale bar, 100 μm. **(B)** Fluorescence intensity analysis of LC3B in oocytes. **(C)** Fluorescence staining of early apoptosis. Scale bar, 100 μm. **(D)** Fluorescence intensity analysis of early apoptosis. All experiments were performed with at least three biological replicates and the data represent the means ± SEMs. *P < 0.05, **P < 0.01, ns = no significance.

Previous studies indicated that *Drp1*, *Fis-1* were related to fission and *Opa1*, *Mfn1*, *Mfn2* were involved in fusion regulation (Zhuan et al., 2022; Xu et al., 2024). First, the expression levels of these genes were analyzed, and a significant decrease in gene expression was found after Atf5 knockdown, when compared to the F_NC group (Figure 3C). Next, increased autophagy level was observed after Atf5 knockdown (402.00 ± 11.36 vs. 463.40 ± 19.68, *P* < 0.01), according to immunofluorescence labeling with LC3B antibody (Figures 4A, B). Furthermore, Annexin V was used to detect early apoptosis in oocytes, and there was no significant difference between the two groups (335.90 ± 21.05 vs. 333.80 ± 23.07, *P* > 0.05; Figures 4C, D). Our results demonstrated that Atf5 knockdown impaired mitochondrial function.

### Vitrification induced mitochondrial dysfunction and triggered mtUPR

**FIGURE 5:**
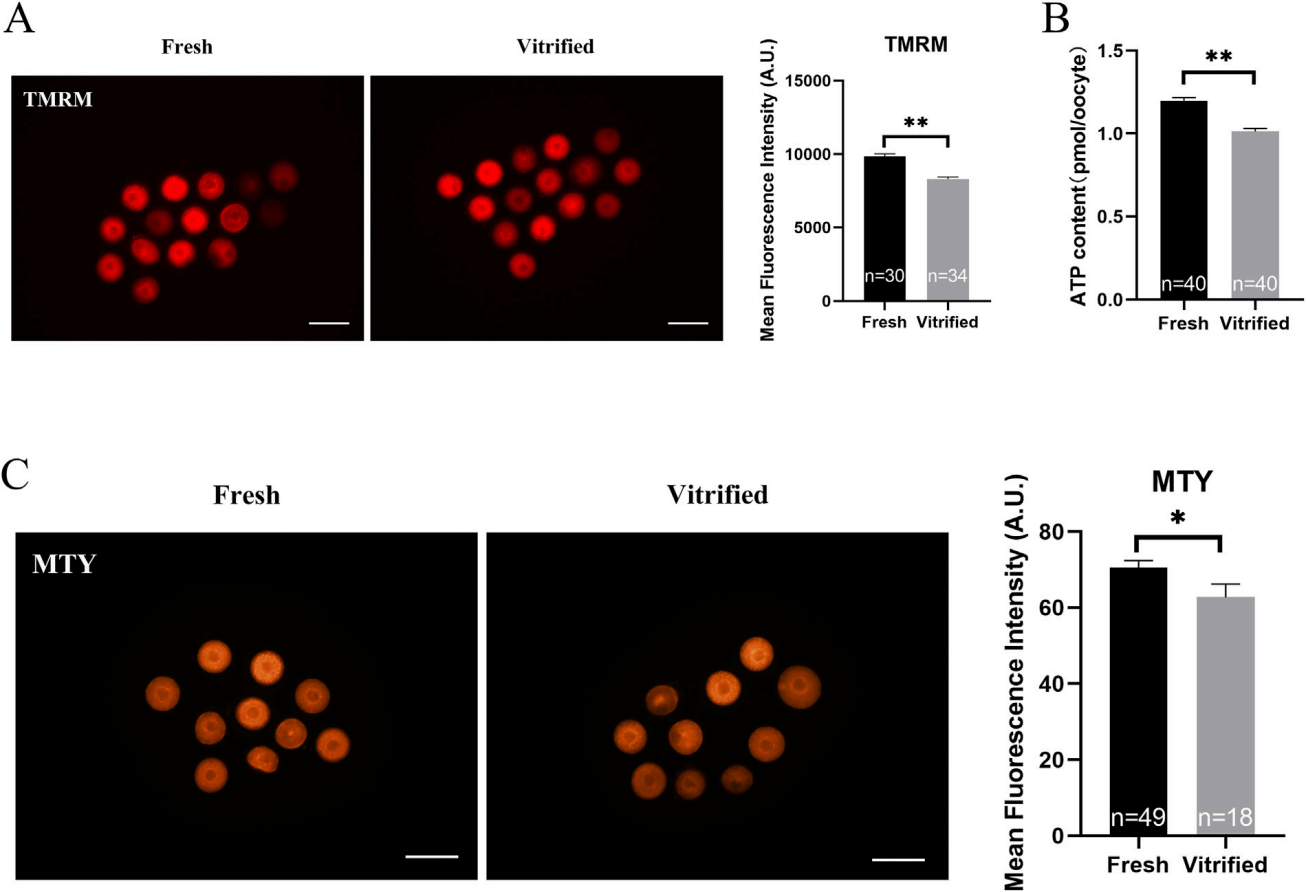
Potential of the mitochondrial membrane, ATP levels and mitochondrial temperature of GV stage oocytes following vitrification. **(A)** Potential of the mitochondrial membrane in GV stage oocytes upon freezing. Scale bar, 100 μm. **(B)** ATP levels in GV stage oocytes after vitrification. **(C)** GV stage oocytes’ mitochondrial temperature upon vitrification. Scale bar, 100 μm. All experiments were performed with at least three biological replicates and the data represent the means ± SEMs. *P < 0.05, **P < 0.01, ns = no significance.

**FIGURE 6:**
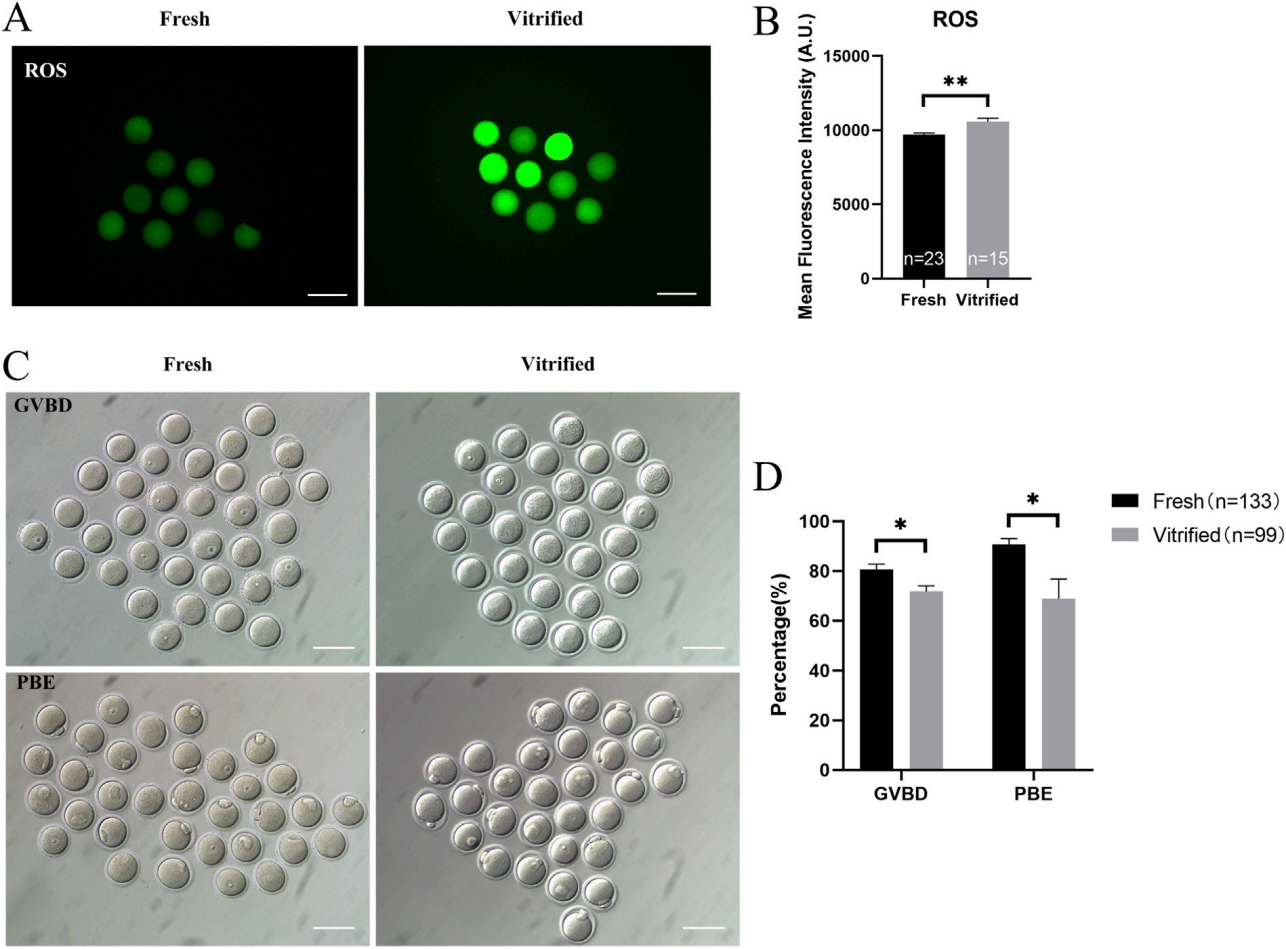
ROS levels and *in vitro* maturation ability of GV stage oocytes following vitrification. **(A, B)** ROS levels in GV stage oocytes after vitrification. Scale bar, 100 μm. **(C, D)** *In vitro* maturation of GV stage oocytes after vitrification. Scale bar, 100 μm. All experiments were performed with at least three biological replicates and the data represent the means ± SEMs. *P < 0.05, **P < 0.01, ns = no significance.

The vitrified group had a much lower mitochondrial membrane potential (9838.00 ± 205.90 vs. 8350.00 ± 162.50, *P* < 0.01; Figure 5A) and ATP levels (1.20 ± 0.02 vs. 1.01 ± 0.02, *P* < 0.01; Figure 5B), whereas the mitochondrial temperature (70.57 ± 1.79 vs. 62.77 ± 3.47, *P* < 0.05; Figure 5C) and ROS levels (9690.00 ± 111.90 vs. 10567.00 ± 245.20, *P* < 0.01; Figures 6A, B) were significantly higher. At the same time, the GVBD rate (80.64 ± 2.15 vs. 71.80 ± 2.21, *P* < 0.05) and PBE rate (90.74 ± 2.32 vs. 68.86 ± 7.93, *P* < 0.05) decreased dramatically (Figures 6C, D).

**FIGURE 7:**
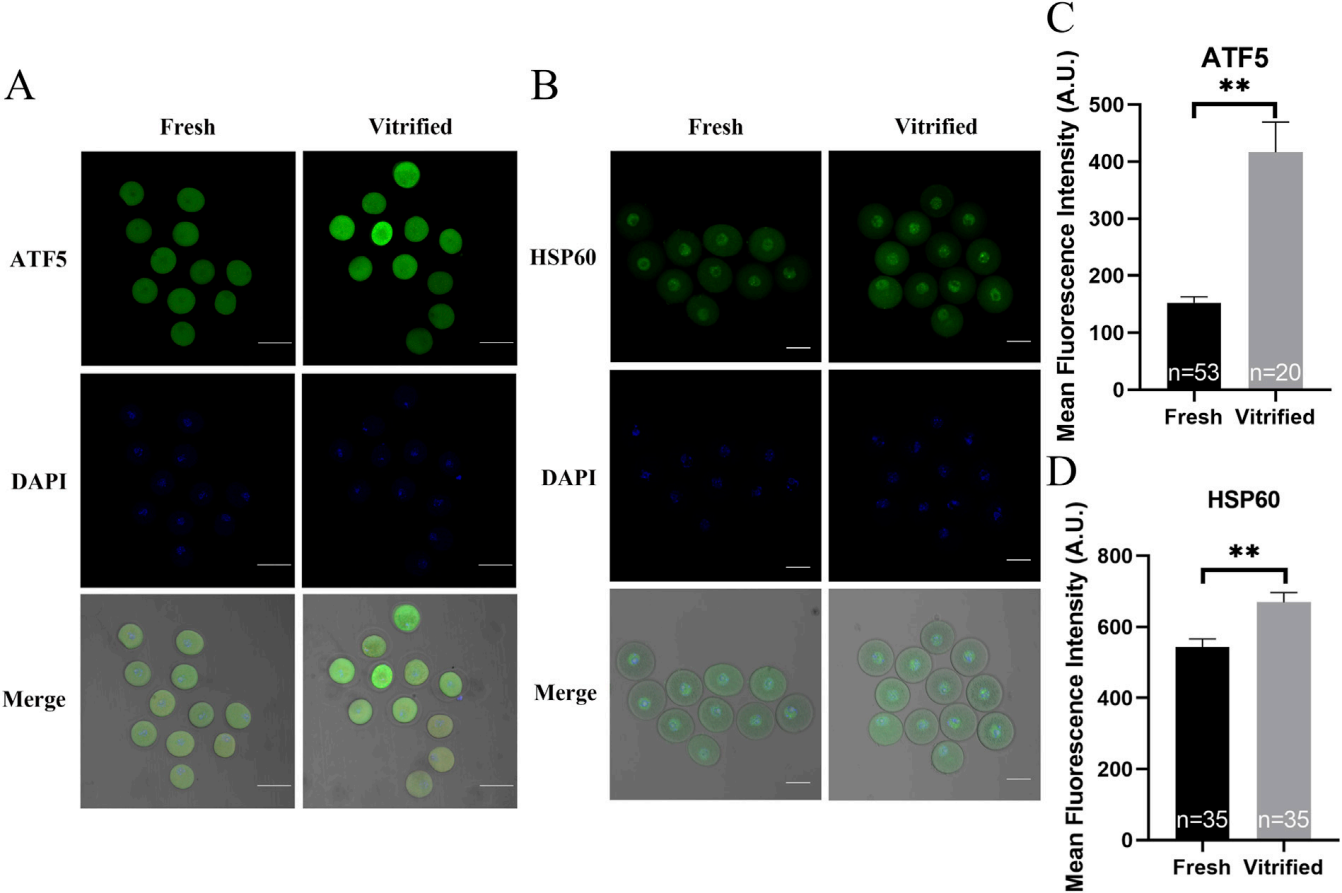
The effects of cryopreservation of GV stage oocytes on the immunofluorescence staining of ATF5 and the mtUPR markers HSP60. **(A, C)** Immunofluorescence staining of ATF5. Scale bar, 100 μm. **(B, D)** Immunofluorescence staining of the mtUPR markers HSP60. Scale bar, 50 μm. All experiments were performed with at least three biological replicates and the data represent the means ± SEMs. *P < 0.05, **P < 0.01, ns = no significance.

mtUPR is a well-studied adaptive process that occurs in the presence of mitochondrial injury (Kim and Sieburth, 2020). The expression of ATF5—one of the main effectors of mtUPR—was significantly higher in the vitrified oocytes (152.40 ± 10.34 vs. 416.40 ± 53.08, *P* < 0.01; Figures 7A, C). To determine whether mtUPR occurs after vitrification, we examined the expression of HSP60, the biomarker for mtUPR. Immunofluorescence labeling results revealed that HSP60 expression was significantly higher in the vitrified group, compared to that in the fresh group (543.90 ± 22.54 vs. 669.10 ± 27.24, *P* < 0.01; Figures 7B, D), indicating that mtUPR was triggered by vitrification. Our findings indicate that vitrification can impair mitochondrial function, decrease the *in vitro* maturation potential of oocytes, and induce mtUPR.

## Lack of Atf5 restored mitochondrial function in vitrified oocytes

**FIGURE 8:**
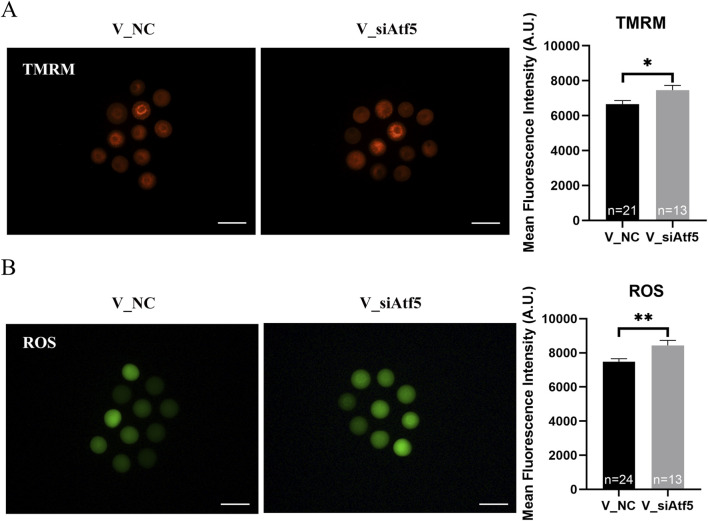
Atf5 knockdown impairs potential of the mitochondrial membrane and ROS levels in vitrified oocytes at GV stage. **(A)** Potential of the mitochondrial membrane after Atf5 knockdown in vitrified oocytes. Scale bar, 100 μm. **(B)** ROS levels after Atf5 knockdown in vitrified oocytes. Scale bar, 100 μm. All experiments were performed with at least three biological replicates and the data represent the means ± SEMs. *P < 0.05, **P < 0.01, ns = no significance.

**FIGURE 9:**
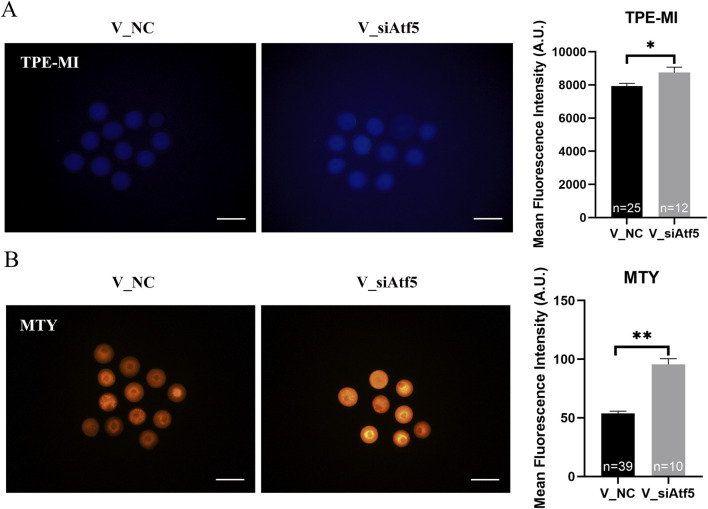
Atf5 knockdown impairs the unfolded proteins and mitochondrial temperature in vitrified oocytes at GV stage. **(A)** TPE-MI dye can be used to detect the unfolded proteins after Atf5 knockdown in vitrified oocytes. Scale bar, 100 μm. **(B)** Mitochondrial temperature upon Atf5 knockdown in vitrified oocytes. Scale bar, 100 μm. All experiments were performed with at least three biological replicates and the data represent the means ± SEMs. *P < 0.05, **P < 0.01, ns = no significance.

**FIGURE 10:**
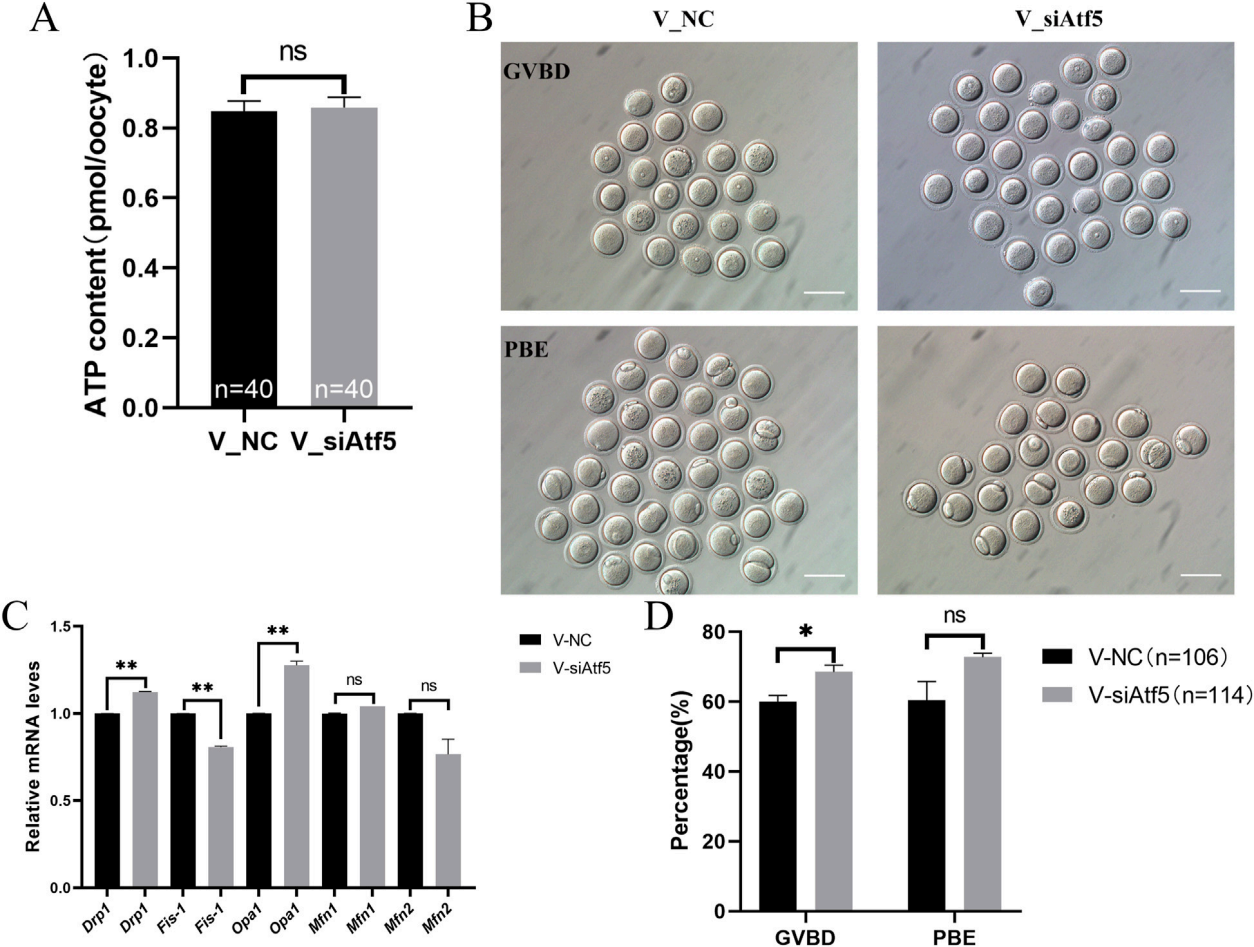
Atf5 knockdown impairs ATP levels, mitochondrial dynamics and *in vitro* maturation ability in vitrified oocytes at GV stage. **(A)** ATP levels after Atf5 knockdown in vitrification. **(B, D)** *In vitro* maturation of GV stage oocytes after Atf5 knockdown in vitrification. Scale bar, 100 μm. **(C)** The relative expression level of mitochondrial dynamic gene mRNA in V_NC vs. V_si. All experiments were performed with at least three biological replicates and the data represent the means ± SEMs. *P < 0.05, **P < 0.01, ns = no significance.

Next, we investigated the role of ATF5 in the mitochondrial function of vitrified oocytes. The mitochondrial membrane potential (6655.00 ± 204.40 vs. 7463.00 ± 268.20, *P* < 0.05; Figure 8A), ROS levels (7483.00 ± 173.80 vs. 8435.00 ± 296.50, *P* < 0.01; Figure 8B), and unfolded proteins levels (7927.00 ± 172.50 vs. 8746.00 ± 325.40, *P* < 0.05; Figure 9A) were much higher in the V_si group. Meanwhile, the mitochondrial temperature was significantly decreased in the V_si group (53.87 ± 1.87 vs. 95.74 ± 4.72, *P* < 0.01; Figure 9B), and ATP levels did not significantly differ between V_NC and V_si groups (0.85 ± 0.03 vs. 0.86 ± 0.03, *P* > 0.05; Figure 10A). In addition, we examined the effects of Atf5 on the *in vitro* maturation capacity of vitrified oocytes, and we found that GVBD in the V_si group was significantly higher than that in the V_NC group (60.02 ± 1.73 vs. 68.52 ± 1.91, *P* < 0.05). There was no statistically significant difference in PBE rate between the two groups (60.39 ± 5.41 vs. 72.77 ± 1.04, *P* > 0.05; Figures 10B, D). These results suggest that Atf5 is involved in mtUPR during oocyte vitrification, and has a positive effect on mitochondrial function and oocyte maturation *in vitro*.

**FIGURE 11:**
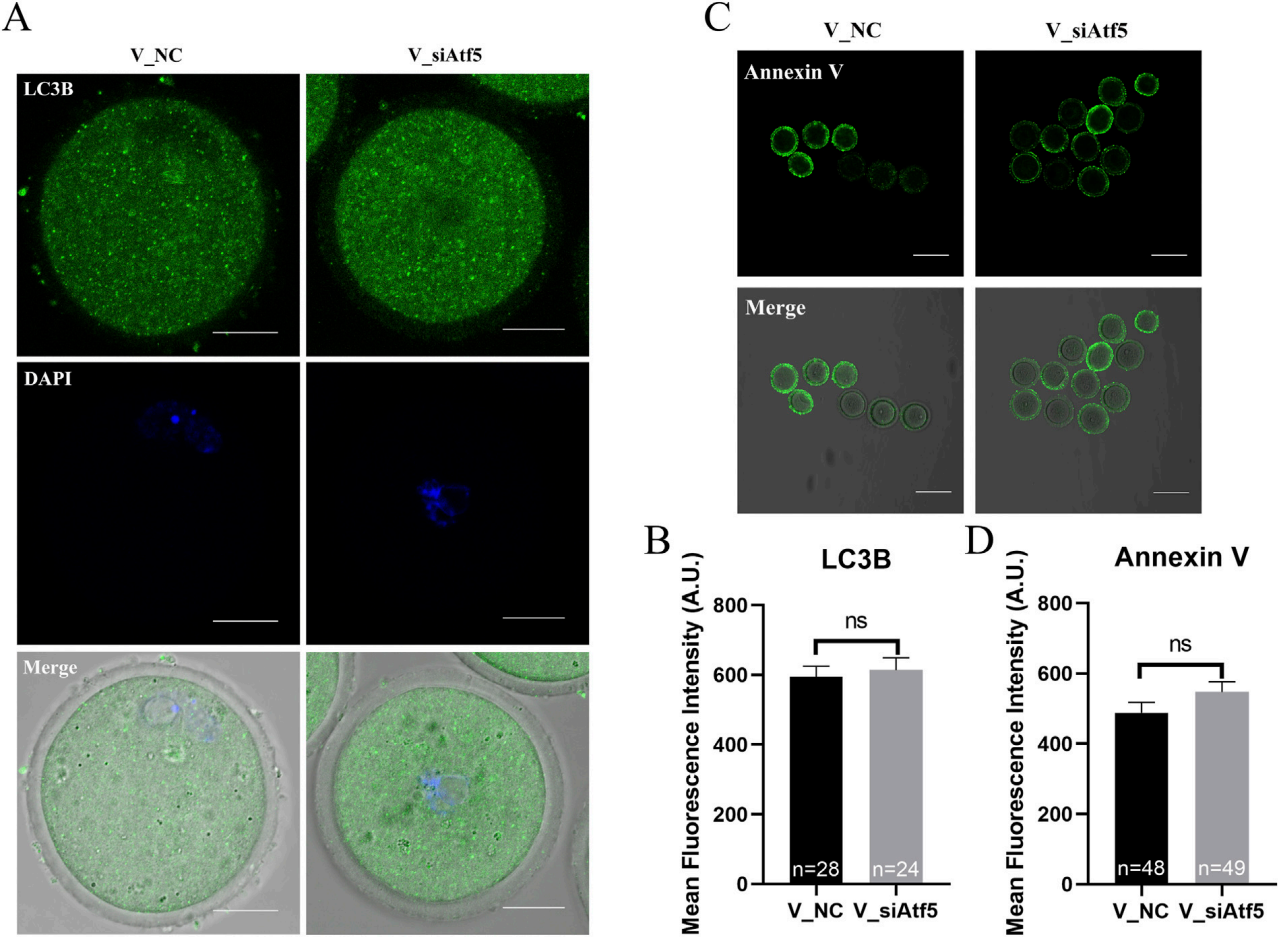
Atf5 knockdown affects autophagy and early apoptosis in GV stage vitrified oocytes. **(A)** Fluorescence staining of autophagy. Scale bar, 100 μm. **(B)** Fluorescence intensity analysis of LC3B in oocytes. **(C)** Fluorescence staining of early apoptosis. Scale bar, 100 μm. **(D)** Fluorescence intensity analysis of early apoptosis. All experiments were performed with at least three biological replicates and the data represent the means ± SEMs. *P < 0.05, **P < 0.01, ns = no significance.

The expression of genes related to fission (*Drp1*) and fusion (*Opa1*) were significantly increased in the V_si group, compared to the V_NC group (Figure 10C). Autophagy levels (595.00 ± 29.95 vs. 614.60 ± 34.53, *P* > 0.05; Figures 11A, B) and early apoptosis levels (488.50 ± 28.98 vs. 547.90 ± 28.47, *P* > 0.05; Figures 11C, D) did not significantly differ between the V_NC and V_si groups. These results indicate that Atf5 knockdown affects mitochondrial dynamics in vitrified oocytes, but has no effect on autophagy (Figures 11A, B) or early apoptosis (Figures 11C, D).

### Systematic analysis of the role of ATF5 in vitrified oocytes by RNA-seq

The biological function of ATF5 in response to vitrification was exploited using RNA-seq. The sequencing data are presented in Supplementary Table S1. Gene expression levels were measured using the commonly used FPKM (fragments per kilobase of exon model per million mapped reads) (Mortazavi et al., 2008). FPKM statistical analysis revealed that the genes in each group were consistent with their distribution range (Supplementary Figure S1A), and there was a good association (with correlation coefficient ranging from 0.9 to 1) between the V_NC and V_si groups of each individual (Supplementary Figure S1B).

**FIGURE 12:**
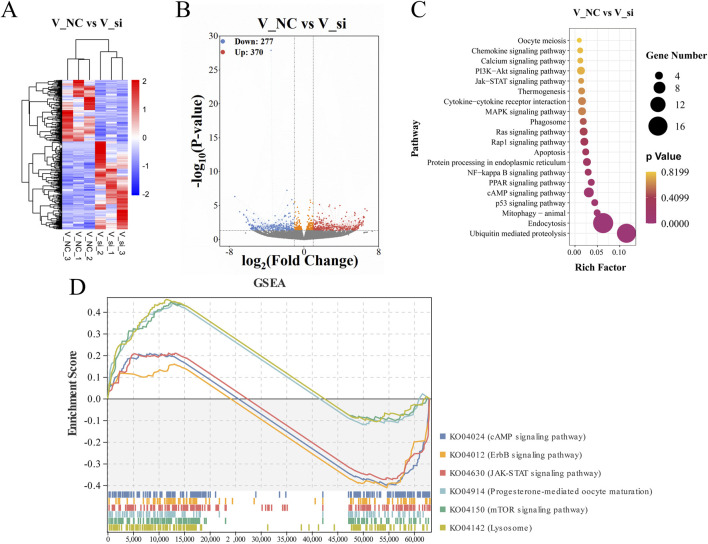
Analysis of gene associations that differ between groups and KEGG enrichment pathway. **(A)** Hierarchical clustering heat map representing differential expression by FPKM cluster analysis. **(B)** A volcanic plot showing the genes that differ between groups, with red representing upregulated genes and blue representing downregulated genes. **(C)** V_NC vs. V_si KEGG enrichment scatterplot. **(D)** V_NC vs. V_si KEGG enrichment pathway in GSEA.

Moreover, differential gene expression cluster analysis was conducted in order to study the expression patterns of differential genes under different treatment conditions after selecting |log2(FoldChange)| > 1.5 and *P*-value < 0.05 as thresholds for differential genes (Figure 12A). We constructed differential volcano maps for the V_NC and V_si groups with the overall distribution of differential genes. In the V_NC vs. V_si group comparison, a total of 370 genes were significantly upregulated, while 277 genes were significantly downregulated (Figure 12B). The top 15 differentially expressed genes are shown in the Supplementary Table S2.

### Functional enrichment analysis

GO and KEGG enrichment analysis were conducted to investigate the biological roles of differentially expressed genes. For the GO enrichment analysis, the biological processes associated to the differential gene enrichment were the positive regulation of cell population proliferation and positive regulation of MAP kinase (MAPK) activity. The molecular functions of differential gene enrichment were transmembrane transporter activity and oxidoreductase activity (Supplementary Figure S1C). The GO-enriched term details of differential genes are shown in Table 1. For KEGG enrichment analysis, the upregulated genes were primarily enriched in the ubiquitin mediated proteolysis, endocytosis pathway, and MAPK signaling pathway, while the downregulated genes were primarily enriched in the cAMP signaling pathway, Ras signaling pathway, and Rap1 signaling pathway (Figure 12C). Supplementary Table S3 shows the KEGG enrichment pathways for each set of differential genes.

**TABLE 1:** The differential expression of mRNAs in V_NC vs. V_si was significantly enriched.

GO term	GO term	V_NC vs. V_si_up	V_NC vs. V_si_down
Biological processes	Positive regulation of proteasomal protein catabolic process	*Rnf144b*	*Rad23a, Osbpl7, Rnf144a, Kcne2*
	Mitotic spindle microtubule depolymerization	*Ccsap*	
	Negative regulation of endocytosis	*Lgals3, Abca2*	*Snph*
	Positive regulation of cell population proliferation	*Cd46, Pax3, Col18a1, Map3k3, Sulf1, Cthrc1, Slc25a33, Ccne1, Zfp703, Frs2, Rogdi*	*Rela, Mmp12, Nrg1, Osmr, Mab21l1, Fgfr1, Tslp, Hbegf, Prl2c5, Anxa1, Ptprn*
	Mitochondria-nucleus signaling pathway	*Slc25a33*	
	Regulation of transcription involved in G2/M transition of mitotic cell cycle	*Bach1*	
	Second-messenger-mediated signaling	*Mc5r, Cxcr6, Pde11a, Cbs*	*Nrg1, Pde10a, Galr1, Fshr, Grin2a, Gprc6a, Taar1*
	cAMP-mediated signaling	*Pde11a, Mc5r*	*Pde10a, Fshr, Taar1, Galr1*
	Positive regulation of MAP kinase activity	*Frs2, Zeb2, Map3k3, Gdf15, Eif2ak2*	*Nrg1, Fgfr1*
Molecular function	cGMP-stimulated cyclic-nucleotide phosphodiesterase activity	*Pde11a*	*Pde10a*
	Transmembrane transporter activity	*Sec61a2, Gabrg3, Slc16a5, Slc9a7, Abca2, Abca17, Kcnab2, Slc16a9, Slc25a33, Gabrq*	*Abcb5, Kcnip4, Slc17a2, Calhm6, Kcne2, Slc22a27, Grin2a*
	Structural constituent of chromatin	*Hmga1b*	
	Potassium channel regulator activity	*Kcnab2*	*Kcne2, Kcnip4*
	Ligand-gated ion channel activity	*Gabrg3, Gabrq*	*Grin2a, Kcne2*
	Oxidoreductase activity	*Decr1, Cyp4a10, Cbs, P4ha2, Kcnab2, Hsd11b1, Cyp2c67, Cyp39a1*	*Bco1, Dus4l, Bbox1*

In addition, we conducted GSEA studies based on common genetic patterns. The most enriched pathways were the lysosomal signaling pathway, mTOR signaling pathway, and cAMP signaling pathway (Figure 12D), which are regulated by the genes *Galc*, *Rras*, *Fshr*, *Pde10a*, *Mapk1,* and so on, in oocyte development following ATF5 knockdown in the context of vitrification (Supplementary Figure S2).

**FIGURE 13:**
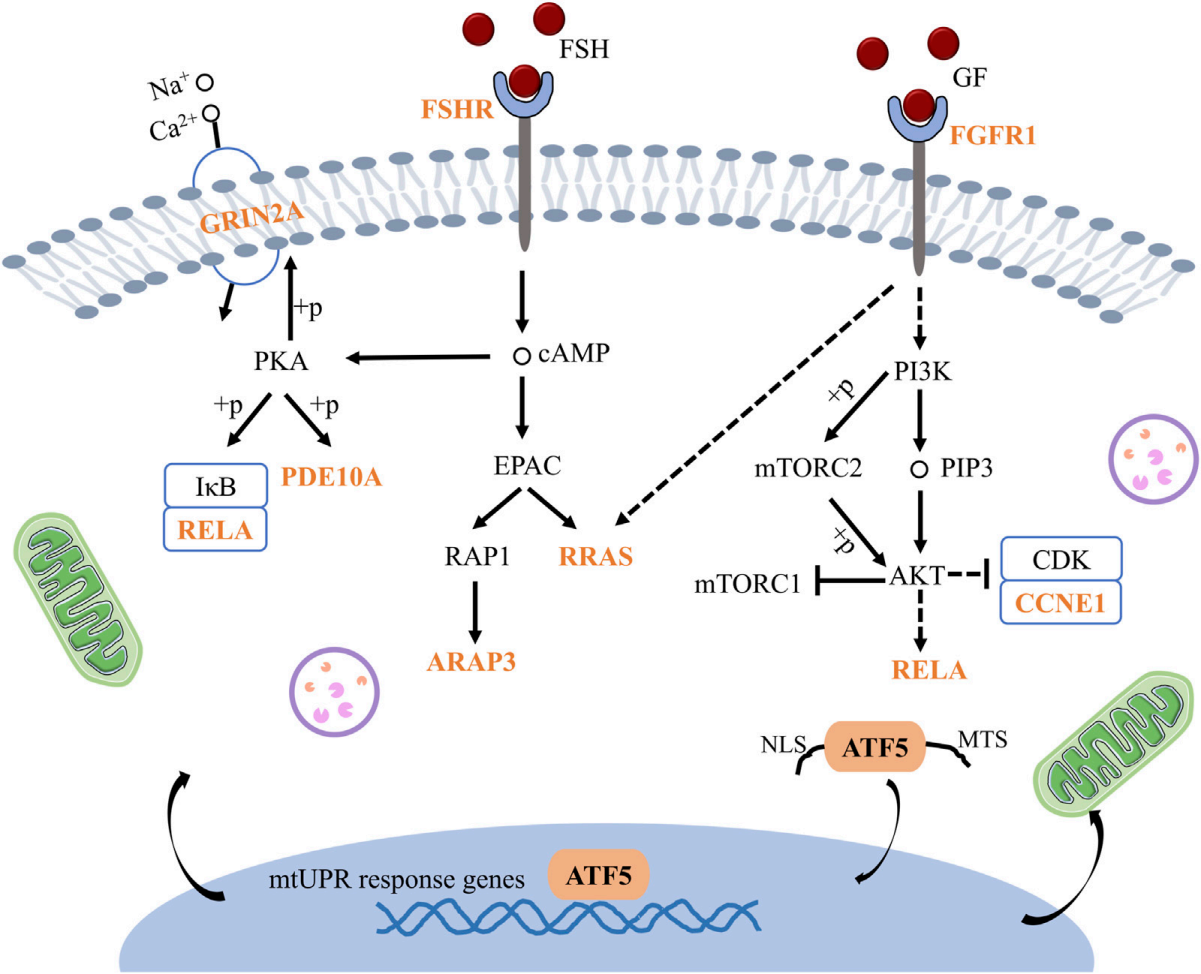
Schematic illustration of the mechanism by which ATF5 modulates oocytes development in vitrified oocytes. GRIN2A: Glutamate receptor, ionotropic, NMDA2A (epsilon 1), PDE10A: Phosphodiesterase 10A, RELA: V-rel reticuloendotheliosis viral oncogene homolog A (avian), FSHR: Follicle stimulating hormone receptor, RRAS: Related RAS viral (r-ras) oncogene, ARAP3: ArfGAP with RhoGAP domain, ankyrin repeat and PH domain 3, FGFR1: Fibroblast growth factor receptor 1, CCNE1: Cyclin E1.

Subsequently, Cytohubba and gProfiler enrichment analysis of GO, KEGG, and GSEA genes revealed that they were mainly enriched in the cAMP-PKA and PI3K/AKT pathways. Finally, we obtained the schematic illustration of the role of ATF5 in GV-stage vitrified oocytes (Figure 13).

## Discussion

ATF5 is an important regulator of mtUPR, a mitochondrial adaptive mechanism which maintains mitochondrial protein homeostasis through activating the transcriptional program of nuclear-encoded mitochondrial chaperones and proteases (Zhu et al., 2021). Accumulating evidence has indicated that disrupted mitochondria are mainly responsible for the decreased *in vitro* maturation potential of oocytes under vitrification stress (Gao et al., 2017). This aroused intriguing questions regarding the role that ATF5 plays in oocytes under vitrification stress.

First, we investigated the influence of ATF5 on mitochondrial function in vitrified oocytes. We demonstrate that vitrification impairs mitochondrial function and inhibits oocyte maturation *in vitro*, which is consistent with previous findings (Wu et al., 2019; Zhuan et al., 2022). Moreover, we discovered that ATF5 expression was aberrantly elevated upon vitrification stress. Since ATF5 played a pivotal role in mtUPR regulation, we evaluated whether mtUPR was initiated by increased ATF5 in vitrified oocytes. As previously reported, HSP60 expression was examined as a marker for mtUPR (Keerthiga et al., 2021; Zhou et al., 2021). Our results showed that HSP60 levels were considerably elevated in vitrified oocytes, suggesting that mtUPR was triggered by vitrification. This is consistent with the findings from hydrogen-induced mtUPR, which promotes the upregulation of mtUPR-associated molecules (ATF5, HSP60) (Hasegawa et al., 2022). Our results further confirm that cells attempt to manage the folding environment and mitochondrial function to maintain mitochondrial protein homeostasis when exposed to stresses (Haynes et al., 2010; Münch, 2018; Cincotta et al., 2022).

Next, the function of ATF5 in protecting oocytes against cryoinjuries was systematically investigated. After Atf5 knockdown, the mitochondrial membrane potential and unfolded protein levels were increased, whereas the mitochondrial temperature decreased in vitrified oocytes. Following the activation of mtUPR in stress granules, ATF5 participates in the PI3K/mTOR signaling pathway, producing an increase in mitochondrial membrane potential, thus boosting cell survival (Lin et al., 2024). The findings are consistent with those of the current investigation (Lin et al., 2024). Some researchers have investigated the effects of varying doses of grape pomace extract on heat-induced oxidative stress and ROS buildup in *C. elegans*, and demonstrated that low and, up to degree ROS levels are favorable and compensate for the effects of heat (Ayuda-Durán et al., 2019). Meanwhile, ROS buildup can decouple the mitochondrial electron transport chain (Wang et al., 2022). Unfolded proteins in vitrified oocytes increase after ATF5 knockdown, which could be attributed to the accumulation of unfolded proteins during vitrification, promoting mtUPR occurrence. These findings are consistent with pervious report that protein folding regulation plays a pivotal role in protein function and homeostasis preservation (Münch, 2018). Furthermore, MTY is a temperature-sensitive fluorescent probe which exhibits a negative correlation between fluorescence intensity and temperature. The metabolic rate can be controlled by modulating mitochondrial temperature, and a slight reduction in mitochondrial temperature may allow the body to maintain its essential physiological processes (Joy et al., 2013), thus maintaining the *in vitro* maturation potential of oocytes. mtUPR is designed to restore mitochondrial protein homeostasis in response to cellular/mitochondrial stress (Kirstein-Miles and Morimoto, 2010; Seli et al., 2019). Protein homeostasis in the mitochondria is restored through mitochondrial fusion/fission or mitophagy (Naresh and Haynes, 2019; Tran and Van Aken, 2020; Vazquez-Calvo et al., 2020). However, long-term activation of the mtUPR causes mitochondrial dysfunction and apoptosis (Tian et al., 2016). In our study, the expression levels of the mitochondrial fission (*Drp1*) and fusion (*Opa1*) genes were increased in vitrified oocytes injected with ATF5 siRNA. It was found that over-expressed mitochondrial fission gene *Drp1* activated mtUPR in skeletal muscle (Touvier et al., 2015). Consistent with previous findings, our results indicated that alterations in mitochondrial dynamics were linked with mtUPR activation. Simultaneously, the GVBD rate was found to increase after Atf5 knockdown in vitrified oocytes during IVM, restoring mitochondrial function through activation of mtUPR, consistent with a previous study reporting that improving protein folding and degradation to maintain protein homeostasis has an impact on development (Haynes and Ron, 2010). At the same time, in order to degrade unfolded proteins, there may be some mechanisms—such as the chaperone system, protease system, and so on—that can modify the state of proteins and increase their stability in cells, allowing cells or mitochondria to maintain protein homeostasis (Hartl et al., 2011; Kwon et al., 2014; Zhang N. J. et al., 2020).

Finally, RNA-seq analysis was carried out to investigate the biological function of ATF5 in vitrification. We discovered that the cAMP-PKA and PI3K/AKT signaling pathways were involved in vitrified oocytes after Atf5 knockdown. A previous report has indicated that accumulated mitochondrial precursor proteins activate mtUPR through the DNAJA1-HSF1 pathway and that HSF1 may be the target gene for ATF5 (Sutandy et al., 2023). Meanwhile, mTORC1 connects mitochondrial stress with mtUPR activation and mitochondrial function restoration through lysosomal-mediated phosphorylation (Li et al., 2023). In addition, some investigations have revealed that ATF5 modulates the stress response via the PI3K/mTOR pathway in stress granules (Lin et al., 2024). In pathological disorders, the SIRT1/ATF5 axis can activate mtUPR, and the expression of chaperones such as HSP60 and HSP10 can be enhanced, reducing mitochondrial dysfunction and oxidative stress injury (Zhao et al., 2023). Furthermore, mTORC2 regulates CMP/GMP proliferation in cells through its involvement in regulating the AKT-Foxo4-ATF5-mtUPR pathway (Shi et al., 2024). These findings are consistent with the biological activity of ATF5 in vitrified oocytes, supporting the significance of the main genes examined in this study.

In summary, our results indicated that decreased ATF5 level contributes to improved mitochondrial function in oocytes exposed to vitrification stress. The present study lays a foundation for deciphering the stress response mechanism of oocytes, as well as providing new clues for the protection of oocytes against cryoinjuries.

The limitation of the study is the limited examination of ATF5 function. The effect of ATF5 knockdown on oocyte developmental potential has not been evaluated in the present study. In addition, our findings need to be further confirmed in other species.

## Materials and methods

### Obtain GV stage oocytes from ICR mice

Female 6-week-old ICR strain mice were purchased from Beijing Weitonglihua Experimental Animal Technology Co., Ltd. Mice were maintained on a 12-h dark/12-h light cycle and were allowed to consume food and water *ad libitum*. After a one-week acclimatization period in the experimental animal room, the mice were used for experiments. The germinal vesicle (GV) stage oocytes were collected according to our previous methods (Zhuan et al., 2022). Briefly, mice were superovulated with 10 IU of pregnant mare serum gonadotropin, and cumulus-oocyte complexes (COCs) were collected after 48 h. GV stage oocytes were obtained from COCs by removing the cumulus cells using mouth pipetting. The experiments were carried out in accordance with the Principles and Guidelines for the Use of Laboratory Animals of China Agricultural University and approved by the Institutional Animal Care and Use Committee of China Agricultural University (AW01302202-1-2).

### Vitrification-warming of mouse GV stage oocytes

Oocyte vitrification was performed according to our previous studies (Zhuan et al., 2022). Briefly, oocytes were first equilibrated in pre-treatment solution (10% ethylene glycol (EG) (v/v) + 10% dimethyl sulfoxide (DMSO) (v/v)) for 30s, then transferred to a vitrification solution [Dulbecco’s phosphate-buffered saline medium (DPBS) with 30% Ficoll (w/v), 15% EG (v/v) and 15% DMSO (v/v) in 0.5 M sucrose], held for 25s, and added into the narrow end of the open pulled straws (OPS). The straws containing oocytes were then quickly dipped into liquid nitrogen for storage.

Oocyte warming was also conducted as the method previously reported (Zhuan et al., 2022). For thawing, the oocyte carrier was ejected from the straw and quickly immersed in thawing medium (0.5 M sucrose) for 5 min. The follow-up experiments were carried out after thawing.

### Microinjection of mouse oocytes

Short-interfering RNAs: All short-interfering RNAs (siRNAs) were purchased from GenePharma. For Atf5 knockdown, three siRNA pairs were created and the negative control siRNA was used as a control. All siRNA sequences are listed in Supplementary Table S4.

As previously mentioned, mouse oocytes were microinjected with 10–15 pL of siRNAs (Jaffe and Terasaki, 2004; Schuh and Ellenberg, 2007), which were diluted to 20 μM. Mouse oocytes at the GV stage were injected with approximately 10 pL of siRNAs using a FemtoJet 4i microinjector (Eppendorf). To fully inhibit endogenous ATF5 expression, injected oocytes were cultured in M2 medium supplemented with 2.5 μM milrinone at 37°C and 5% CO_2_ in air for 24 h as previously reported (Zhang L. et al., 2020). The F_NC and F_si groups were negative control and siAtf5 in fresh oocytes, respectively. The V_NC and V_si groups were negative control and siAtf5 in vitrified oocytes after siRNA, respectively. The knockdown efficiency of Atf5 is shown in Supplementary Figure S3A. Finally, the siAtf5-722 sequence was selected for future study. The experimental design in Supplementary Figure S3B.

### RNA sequencing (RNA-seq) analysis

The RNA-Seq method was used to amplify each sample (20 GV stage oocytes for each group), according the manufacturer’s instructions. A total of six samples from two conditions were used for total RNA extraction and library construction.

Quantification of gene expression levels and differential expression analysis: Gene-specific read counts for RNA-seq data were calculated using HTSeq-count (v0. 5.4). Gene expression levels were estimated as fragments per kilobase of transcript per million fragments mapped (FPKM). Differential gene expression analysis was performed with the DESeq R package (1.10.1), which was used for differential gene expression analysis with respect to the vitrified group (V_NC, V_siAtf5).

GO and KEGG enrichment analysis and gene interaction analysis: Gene Ontology enrichment analysis was performed using the Gene Ontology Consortium enrichment analysis tool (Young et al., 2010). We used the KOBAS software (Mao et al., 2005) to test the statistical enrichment of differential expression genes in KEGG signaling pathways. Genetic interaction analysis was performed at https://www.string-db.org/.

Gene set enrichment analysis (GSEA) was performed using the GSEA software (version 3.0), which utilizes pre-defined gene sets from the Molecular Characterization Database (version 6.0).

### Quantitative real time polymerase chain reaction (qRT-PCR)

RNA was extracted after treatment using the TRIzol chloroform extraction method, following which it was converted into cDNA using PrimeScript™ RT Master Mix (Perfect Real Time; Cat. RR036A, Takara). The primers were tested for efficiency to ensure their specificity. qRT-PCR was performed using TransStart^®^ Tip Green qPCR SuperMix (+Dye II) (Cat. AQ142-22, Transgen). qRT-PCR was performed using an ABI 7500 qRT-PCR system (Applied Biosystems). The relative expression levels of the target genes were calculated using the 2^−ΔΔCt^ method. Peptidylprolyl isomerase A (*Ppia*) and ribosomal protein L7 (*Rpl7*) were used as reference genes, according to previous reports (Bang et al., 2014; Wang et al., 2021). All primer sequences are listed in Table 2.

**TABLE 2:** Primer sequences used for qRT-PCR.

Gene	Primer sequence	NCBI reference sequence
*Drp1*	F: 5′-CAG​GTG​GTG​GGA​TTG​GAG​AC-3′	NM_001025947.2
	R: 5′-CTG​GCA​TAA​TTG​GAA​TTG​GTT​T-3′	
*Fis-1*	F: 5′-TGT​CCA​AGA​GCA​CGC​AAT​TTG-3′	NM_001163243.1
	R: 5′-CCT​CGC​ACA​TAC​TTT​AGA​GCC​TT-3′	
*Opa1*	F: 5′-CCG​AGG​ATA​GCT​TGA​GGG​TT-3′	NM_001199177.2
	R: 5′-CGT​TCT​TGG​TTT​CGT​TGT​GA-3′	
*Mfn1*	F: 5′-GGA​CTT​TAT​CCG​AAA​CCA​GA-3′	NM_024200.5
	R: 5′-TGA​GAT​TGA​AGA​ATG​GAG​GC-3′	
*Mfn2*	F: 5′-TTC​TTG​TGG​TCG​GAG​GAG​TG-3′	NM_001285920.1
	R: 5′-CTT​TGG​TGG​TCC​AGG​TCA​GT-3′	
*Rpl7*	F: 5′-TCA​ATG​GAG​TAA​GCC​CAA​AG-3′	NM_011291.5
	R: 5′-CAA​GAG​ACC​GAG​CAA​TCA​AG-3′	
*Ppia*	F: 5′-GAG​CTG​TTT​GCA​GAC​AAA​GTT​C-3′	NM_008907.2
	R: 5′-CCC​TGG​CAC​ATG​AAT​CCT​GG-3′	

### Mitochondrial membrane potential detection

The mitochondrial membrane potential was measured using tetramethylrhodamine, methyl ester (TMRM) fluorescent probe (Cat. I34361, Invitrogen). Oocytes were treated with 100 nM TMRM diluted in M2 containing milrinone medium for 30 min at 37°C. After washing three times with M2 containing milrinone medium, images were captured using an Olympus IX71 inverted fluorescence microscope. Fluorescence intensities were measured using the EZ-C1 FreeViewer software and the average fluorescence intensity per unit area within this region of interest (ROI) was determined to quantify the fluorescence of individual oocyte images.

### Determination of the mitochondrial temperature

As previously reported, the mitochondrial temperature was measured using Mito-Thermos Yellow (MTY) fluorescent probe, the fluorescence intensity of which is negatively correlated with temperature (Zhuan et al., 2022). Oocytes were treated with 0.5 μM MTY diluted in M2 containing milrinone medium for 15 min at 37°C. After washing three times with M2 containing milrinone medium, images were captured using an Olympus IX71 inverted fluorescence microscope. Fluorescence intensities were measured using the EZ-C1 FreeViewer software and the average fluorescence intensity per unit area within this ROI was determined to quantify the fluorescence of individual oocyte images.

### Reactive oxygen species (ROS) levels detection

ROS levels were measured using fluorescent probe 2′,7′-dichlorofluorescein diacetates (DCFHDA; Cat. D399, Invitrogen). Oocytes were treated with 1 mM 2′7′-DCFHDA diluted in M2-containing milrinone medium for 30 min at 37°C. After washing three times with M2 containing milrinone medium, images were captured using an Olympus IX71 inverted fluorescence microscope. Fluorescence intensities were measured using the EZ-C1 FreeViewer software and the average fluorescence intensity per unit area within this ROI was determined to quantify the fluorescence of individual oocyte images.

### Determination of the ATP levels

As previously reported, ATP levels were measured using an enhanced ATP assay kit (Cat. S0027, Beyotime) (Zhou et al., 2022). Briefly, each group of oocytes (10 oocytes per sample) was transferred into 50 μL lysis buffer with vortexing to lyse the cells. ATP detection working solution (50 μL/well) was added to a new 96-well plate and allowed to stand at room temperature for 5 min. Five standard wells were set up, and the standard sample (0 μL, 2 μL, 4 μL, 8 μL, 16 μL) was added to a 96-well plate. Then, the volume was brought to 50 μL with lysis buffer. For sample wells, 50 μL of sample lysis buffer was added. ATP levels were determined using an Infinite F200 microplate reader.

### Detection of the unfolded protein levels

Unfolded protein levels were measured by tetraphenylethene maleimide (TPE-MI) staining method according to previous study (Chen M. Z. et al., 2017). The GV-stage oocytes were stained with 100 μL of M2 medium containing milrinone and 0.2 μL of TPE-MI at 37°C for 30 min. Oocytes were washed three times in M2 medium containing milrinone. Images were captured under an IX71 (Olympus) inverted fluorescence microscope. Fluorescence intensities were measured using the EZ-C1 FreeViewer software and the average fluorescence intensity per unit area within this ROI was determined to quantify the fluorescence of individual oocyte images.

### Immunofluorescence staining

The GV-stage oocytes were fixed in an immunofluorescence staining solution (Cat. P0098, Beyotime) in PBS for 30 min at room temperature, permeabilized in 0.1% Triton X-100% and 0.1% poly (vinyl alcohol) (PVA) in PBS for 1 h at room temperature, and blocked in 3% BSA, 0.1% Triton X-100, and 0.1% PVA in PBS (blocking buffer) for 1 h at room temperature. The oocytes were then incubated with anti-LC3B (1:100, Cat. 3868S, Cell signaling technology), ATF5 (1:200, Cat. AF2563, Beyotime), and HSP60 (1:200, Cat. bs-0191R, Bioss) antibody overnight. Images were captured and analyzed using a Nikon A1 confocal microscope.

### Apoptosis levels measurement

The GV-stage oocytes obtained under different treatments were randomly distributed into two groups. An Annexin V staining kit (Cat. A211-01, Vazyme) was used to detect the early apoptosis levels in oocytes. Briefly, oocytes were stained for 10 min with 100 μL of binding buffer containing 5 μL of Annexin-V-FITC at 37°C. Oocytes were washed three times in M2 medium containing milrinone, and images were captured and analyzed using a Nikon A1 confocal microscope.

### *In vitro* maturation (IVM)

The GV-stage oocytes obtained under different treatments were randomly distributed into different groups. The oocytes were washed three times in IVM medium (Cat. M7292, Sigma) and cultured for IVM. And as previously reported, oocytes cultured for 2 h and 12 h were collected to evaluate GVBD and polar body extrusion (PBE) rates, respectively (Zhuan et al., 2022).

### Statistical analysis

All statistical analyses were performed using the GraphPad Prism 8 software. Differences between two groups were assessed using independent t-test and chi-square test. At least three replicates were performed in all experiments, with results expressed as means ± SEMs. A value of *P < 0.05 was considered significant, **P < 0.01 was considered highly significant, and ns was considered as no difference.

## Data Availability

The original contributions presented in the study are included in the Supplementary Material; further inquiries can be directed to the corresponding authors.
