# Supplementary material for: Decreased ATF5 level contributes to improved mitochondrial function in oocytes exposed to vitrification stress
Source: Front Cell Dev Biol. 2024 Sep 20;12:1431683. doi: 10.3389/fcell.2024.1431683 (PMC11449845; doi:10.3389/fcell.2024.1431683)
Supplement: Supplementary file 1 [file DataSheet1.docx]

***Supplementary Material***

**Supplementary Table 1. Summary of sequenced RNA-seq data.**

| **Samples** | **Total reads** | **GC content (%)** | **Total mapped** |
| --- | --- | --- | --- |
| V_NC_1 | 44962550 | 43.59 | 43834604 (97.49%) |
| V_NC_2 | 50442298 | 43.90 | 48940336 (97.02%) |
| V_NC_3 | 54956718 | 43.70 | 53706642 (97.73%) |
| V_si_1 | 39145938 | 44.36 | 38169344 (97.51%) |
| V_si_2 | 42161510 | 43.56 | 41388770 (98.17%) |
| V_si_3 | 50780916 | 43.57 | 49681866 (97.84%) |
| Total | 282449930 | - | 275721562(97.62%) |

The V_NC and V_si groups received negative control and siAtf5, respectively.

**Supplementary Table 2. Differential expression up-and down-regulated mRNAs top 15 in V_NC vs. V_si.**

| **Gene ID** | **Gene symbol** | **Description** | ***p*-value** | **Fold Change** | | **Up/Down** |
| --- | --- | --- | --- | --- | --- | --- |
| ENSMUSG00000045268 | Zfp691 | Zinc finger protein 691 | 7.57E-06 | | 1.6737 | Up |
| ENSMUSG00000075574 | Defb48 | Defensin beta 48 | 1.00E-05 | | 3.8512 | Up |
| ENSMUSG00000074704 | Rad21l | RAD21-like (S. pombe) | 1.45E-05 | | 2.6878 | Up |
| ENSMUSG00000023882 | Zfp54 | Zinc finger protein 54 | 3.06E-05 | | 6.5381 | Up |
| ENSMUSG00000016918 | Sulf1 | Sulfatase 1 | 9.58E-05 | | 4.5646 | Up |
| ENSMUSG00000032649 | Colgalt2 | Collagen beta(1-O) galactosyltransferase 2 | 0.00010213 | | 5.4415 | Up |
| ENSMUSG00000031283 | Chrdl1 | Chordin-like 1 | 0.00014106 | | 1.969 | Up |
| ENSMUSG00000056824 | Zfp663 | Zinc finger protein 663 | 0.00027818 | | 5.4472 | Up |
| ENSMUSG00000030223 | Ptpro | Protein tyrosine phosphatase, receptor type, O | 0.00040749 | | 4.47 | Up |
| ENSMUSG00000067649 | Mageb18 | Melanoma antigen family B, 18 | 0.00043787 | | 6.3768 | Up |
| ENSMUSG00000074825 | Itpripl1 | Inositol 1,4,5-triphosphate receptor interacting protein-like 1 | 0.00045093 | | 1.4095 | Up |
| ENSMUSG00000043336 | Filip1l | Filamin A interacting protein 1-like | 0.00064372 | | 6.4063 | Up |
| ENSMUSG00000043252 | Tmem64 | Transmembrane protein 64 | 0.00082021 | | 2.4022 | Up |
| ENSMUSG00000024766 | Lipo3 | Lipase, member O3 | 0.00098586 | | 1.6381 | Up |
| ENSMUSG00000024792 | Zfpl1 | Zinc finger like protein 1 | 0.001347 | | 1.0957 | Up |
| ENSMUSG00000022286 | Grhl2 | Grainyhead like transcription factor 2 | 5.74E-06 | | -1.093 | Down |
| ENSMUSG00000048647 | Exd1 | Exonuclease 3'-5' domain containing 1 | 0.00014117 | | -1.6309 | Down |
| ENSMUSG00000067656 | Slc22a27 | Solute carrier family 22, member 27 | 0.00015883 | | -5.3718 | Down |
| ENSMUSG00000085156 | Snhg15 | Small nucleolar RNA host gene 15 | 0.00049742 | | -1.6141 | Down |
| ENSMUSG00000051354 | Samd3 | Sterile alpha motif domain containing 3 | 0.00052139 | | -6.1728 | Down |
| ENSMUSG00000009628 | Tex15 | Testis expressed gene 15 | 0.00064122 | | -1.0243 | Down |
| ENSMUSG00000017176 | Nt5c3b | 5'-nucleotidase, cytosolic IIIB | 0.00098972 | | -6.0391 | Down |
| ENSMUSG00000022848 | Dirc2 | Disrupted in renal carcinoma 2 (human) | 0.0028587 | | -1.2756 | Down |
| ENSMUSG00000027908 | Tchhl1 | Trichohyalin-like 1 | 0.0029345 | | -6.165 | Down |
| ENSMUSG00000037440 | Vnn1 | Vanin 1 | 0.0029868 | | -5.2368 | Down |
| ENSMUSG00000029088 | Kcnip4 | Kv channel interacting protein 4 | 0.0039253 | | -1.4033 | Down |
| ENSMUSG00000092416 | Zfp141 | Zinc finger protein 141 | 0.0041001 | | -1.0924 | Down |
| ENSMUSG00000006784 | Ttc25 | Tetratricopeptide repeat domain 25 | 0.0042065 | | -2.1438 | Down |
| ENSMUSG00000053856 | Dnajc5g | DnaJ heat shock protein family (Hsp40) member C5 gamma | 0.0042401 | | -1.3247 | Down |
| ENSMUSG00000072791 | Abcb5 | ATP-binding cassette, sub-family B (MDR/TAP), member 5 | 0.0049456 | | -1.19 | Down |

**Supplementary Table 3. mRNAs differentially expressed in V_NC vs. V_si significantly enriched in KEGG pathway.**

| Pathway | V_NC vs. V_si_up | V_NC vs. V_si_down |
| --- | --- | --- |
| Ubiquitin mediated proteolysis | *Mid1, S100a2* | *Birc7* |
| Endocytosis | *S100a2, Ehd3, H2-T-ps* | *Arap3* |
| cAMP signaling pathway |  | *Rela, Grin2a, Rras, Fshr, Arap3, Pde10a* |
| Oocyte meiosis | *Ccne1* |  |
| Thermogenesis | *Frs2, Ndufaf1* | *Fgfr1* |
| Mitophagy - animal |  | *Rela, Rras* |
| NF-kappa B signaling pathway |  | *Rela, Pidd1* |
| Protein processing in endoplasmic reticulum | *Sec61a2, Eif2ak2* | *Rad23a, Dnajc5g* |
| p53 signaling pathway | *Ccne1* | *Pidd1, Perp* |
| PPAR signaling pathway | *Cyp4a10* | *Hmgcs2, Gk2* |
| Ras signaling pathway |  | *Rela, Grin2a, Fgfr1, Rras* |
| Rap1 signaling pathway |  | *Arap3, Grin2a, Fgfr1, Rras* |
| Phagosome | *H2-T-ps, Sec61a2* | *Fcgr3* |
| Jak-STAT signaling pathway |  | *Tslp, Osmr* |
| MAPK signaling pathway | *Map3k3* | *Rela, Fgfr1, Rras* |

**Supplementary Table 4. Nucleotide sequences of Atf5 siRNAs, and of the control siRNA.**

| Gene | siRNA sequence |
| --- | --- |
| Negative Control | 5´-UUCUCCGAACGUGUCACGUTT-3´ |
|  | 5´-ACGUGACACGUUCGGAGAATT-3´ |
| siAtf5*-*722 | 5´-GCCCUUGCCCACCUUUGACTT-3´ |
|  | 5´-GUCAAAGGUGGGCAAGGGCTT-3´ |
| siAtf5*-*820 | 5´-GCUUGUCAACCCUGCCUGUTT-3´ |
|  | 5´-ACAGGCAGGGUUGACAAGCTT-3´ |
| siAtf5-964 | 5´-UCAGGUACCGCCAGAGGAATT-3´ |
|  | 5´-UUCCUCUGGCGGUACCUGATT-3´ |


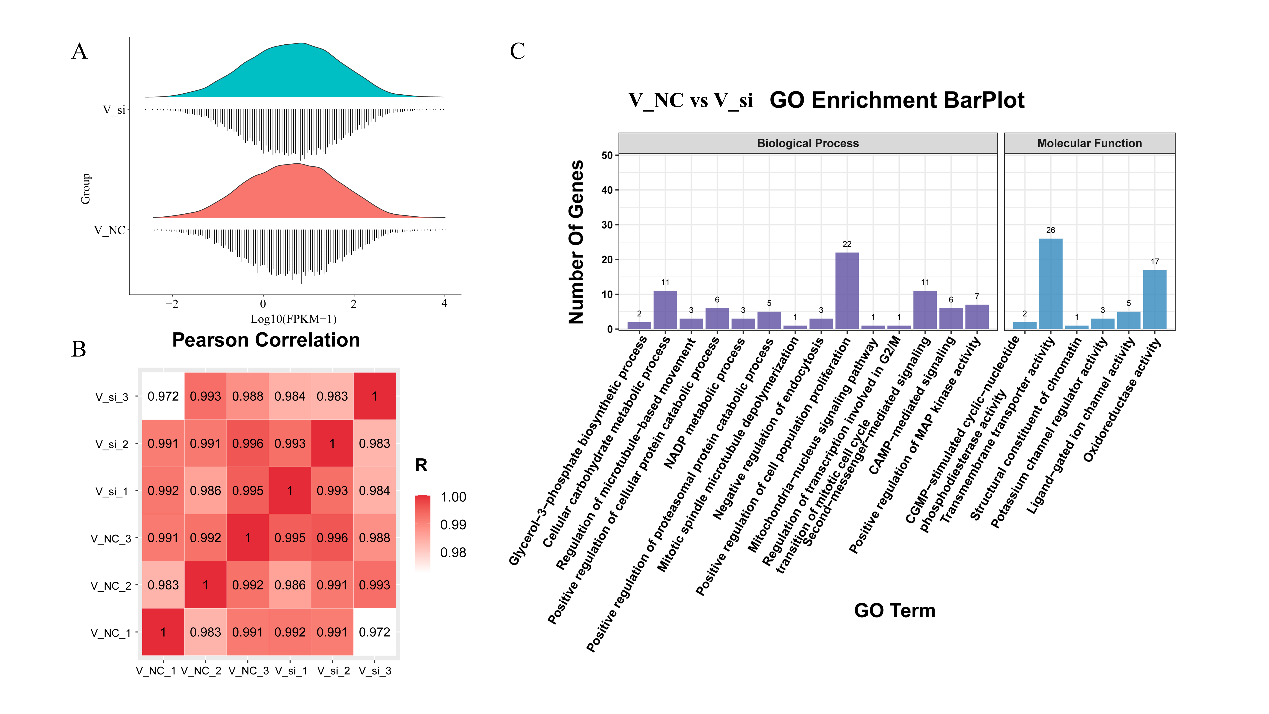


**Supplementary Figure 1.** Quality assessment and the Pearson correlation analysis of RNA-seq data, and GO enrichment analysis. **(A)** FPKM (log10 FPKM+1) density distribution in each sample. **(B)** The correlation plot shows the Pearson correlation between groups. **(C)** V_NC vs. V_si GO enrichment barplot.


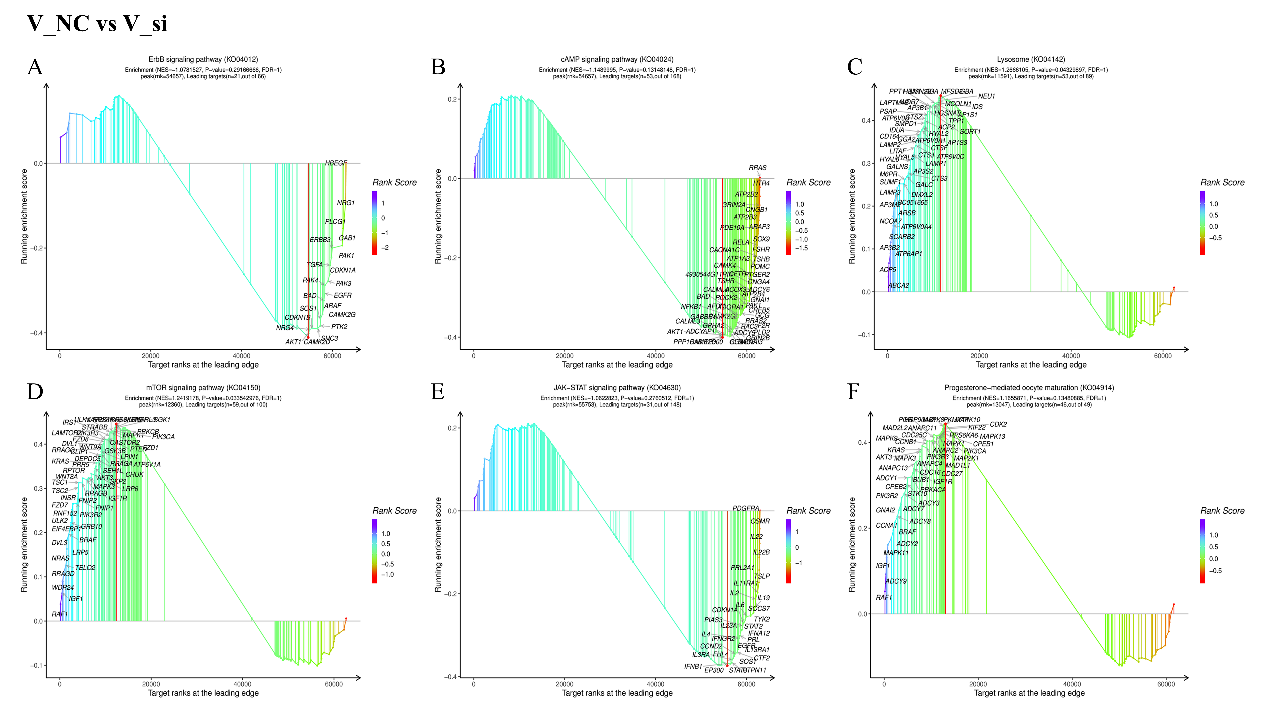


**Supplementary Figure 2.** GSEA bitmap displays gene expression profiles in each pathway for V_NC vs. V_si. **(A)** The gene expressions of ErbB signaling pathway (KO04012) in GSEA analysis. **(B)** The gene expressions of cAMP signaling pathway (KO04024) in GSEA analysis. **(C)** The gene expressions of Lysosome (KO04142) in GSEA analysis. **(D)** The gene expressions of mTOR signaling pathway (KO04150) in GSEA analysis. **(E)** The gene expressions of JAK-STAT signaling pathway (KO04630) in GSEA analysis. **(F)** The gene expressions of Progesterone-mediated oocyte maturation (KO04914) in GSEA analysis.


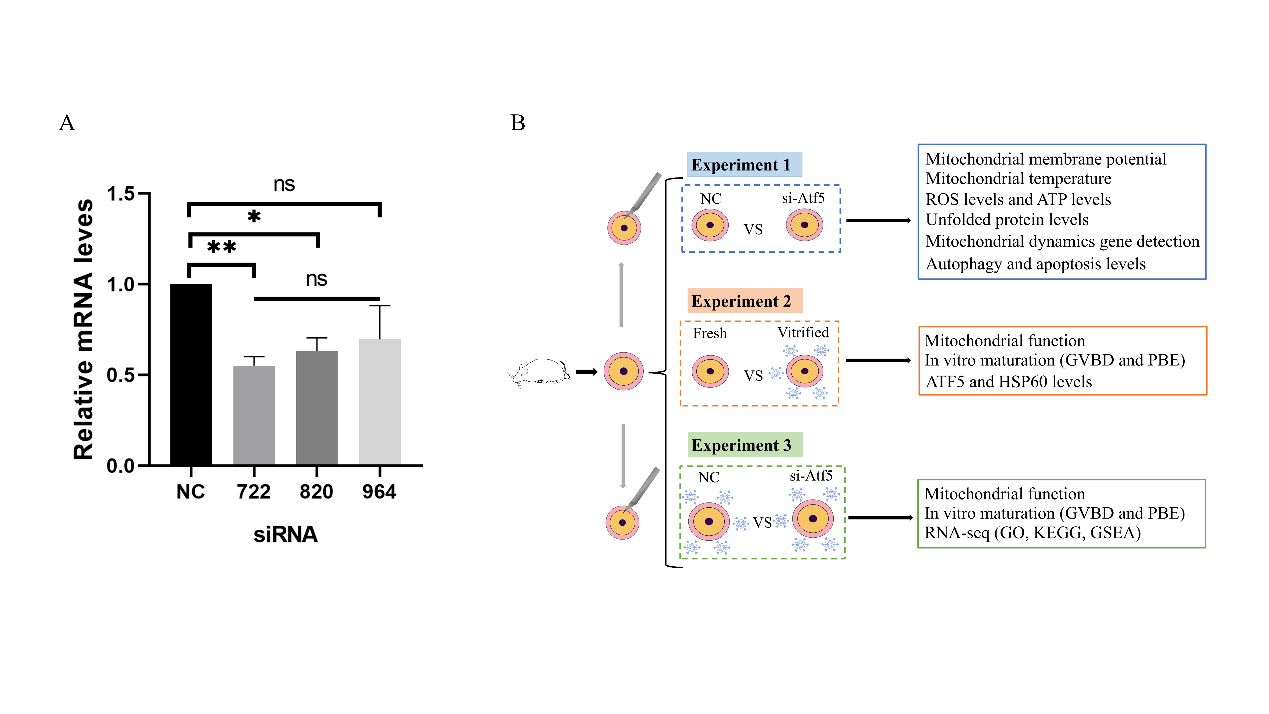


**Supplementary Figure 3.** qRT-PCR verification of Atf5 siRNA knockdown efficiency and the experimental design of this study. **(A)** All experiments were performed with at least three biological replicates and the data represent the means ± SEMs. * *P* < 0.05，** *P* < 0.01, ns=no significance. **(B)** The experimental design of this study.
